# Foreign Correspondence

**Published:** 1890-03

**Authors:** Henry I. Moore

**Affiliations:** 24 Rue Serviez, Pau, S. France


### Foreign Correspondence.

---

To the Editor:

Thinking that cocaine notes may still be interesting, I send you those of three cases that with me lately have deviated from the ordinary run of success.

For a lady patient, twenty-two years of age, I injected ten minims containing in solution one-half grain of cocaine, intending to extract the roots of a lower first molar. The part was entirely insensible, but she, fearing great pain and knowing the instrument about to be applied, made it impossible to operate without force, which I did not attempt. I tried to persuade her, but after five minutes I saw she was getting uneasy and commenced convulsive movements with her hands. They were not cold, neither were the lips or face blanched; the pulse was good, but the breathing rather troubled. After all tight clothing had been loosened and strong smelling-salts used, everything seemed to get natural, and her mother fixed her for leaving; but no sooner had her corsets been fastened than the old trouble reappeared increased. She was again relieved and aroused from a faint, and brandy was given; and I found the best thing was to get her on the floor, where she lay for about an hour in an hysterical fit, breathing jerkily. I called in a doctor, as I had never met with such symptoms before with cocaine, but he pronounced it only hysteria. She awoke all right and left for home.

The second case was a gentleman about fifty years of age. He had been suffering all night from an exposed nerve in an almost invisible distal cavity of a second upper molar, and a doctor, to relieve him, had at different times given him two weak injections of morphia over the mastoid process; but as he got no relief, I was summoned in the morning. I injected half a grain of cocaine, having told him before that I could save the tooth; but no, he wanted it out. However, no sooner did he feel the pain gone than he changed his mind and asked me to treat it. So I put in a dressing of carbolized resin, and was about to leave him, when he complained of sickness, and his face looked distressed and his lips blanched. I had no ammonia with me, as we were in a hotel, so I bathed his face and opened the windows; he seemed set against brandy, so we tried some Chartreuse, which, acting like a charm, speedily relieved him.

The last was a boy of seventeen years. I was injecting cocaine slowly, but no sooner had three minims disappeared, when I found him fainting. He was very anæmic and weak. Later on for him I operated without an anæsthetic.

Looking back over three years' experience with cocaine, I think that where it has been injected, and for some reason no extraction has taken place, and therefore no bleeding, uneasiness may be caused. I find a minute and a half to two minutes ample time to wait, and, used with care and common sense, it has ever proved to me a valuable help.

Henry I. Moore, D.D.S., L.D.S.E.

24 Rue Serviez, Pau, S. France.

